# *catena*-Poly[[bis­(3-acetyl­pyridine-κ*N*)cadmium]-di-μ-seleno­cyanato-κ^2^ *N*:*Se*;κ^2^ *Se*:*N*]

**DOI:** 10.1107/S1600536812018375

**Published:** 2012-04-28

**Authors:** Julia Werner, Jan Boeckmann, Inke Jess, Christian Näther

**Affiliations:** a Institut für Anorganische Chemie, Christian-Albrechts-Universität Kiel, Max-Eyth-Strasse 2, 24118 Kiel, Germany

## Abstract

In the crystal structure of the title compound, [Cd(NCSe)_2_(C_7_H_7_NO)_2_]_*n*_, the Cd^2+^ cation is coordinated by two 3-acetyl­pyridine ligands and four μ-1,3-bridging seleno­cyanate anions within a slightly distorted CdN_4_Se_2_ octa­hedron. The asymmetric units consists of one Cd^2+^ cation, which is situated on a center of inversion, as well as one seleno­cyanate anion and one 3-acetyl­pyridine ligand in general positions. The metal cations are μ-1,3-bridged *via* the seleno­cyanate anions into chains along the *a* axis.

## Related literature

For general background information including details on thermal decomposition reactions and magnetic properties of the precursor and μ-1,3 bridging compounds, see: Näther & Greve (2003 ▶); Boeckmann & Näther (2010 ▶, 2011 ▶); Wöhlert *et al.* (2011 ▶). For a description of the Cambridge Structural Database, see: Allen (2002 ▶).
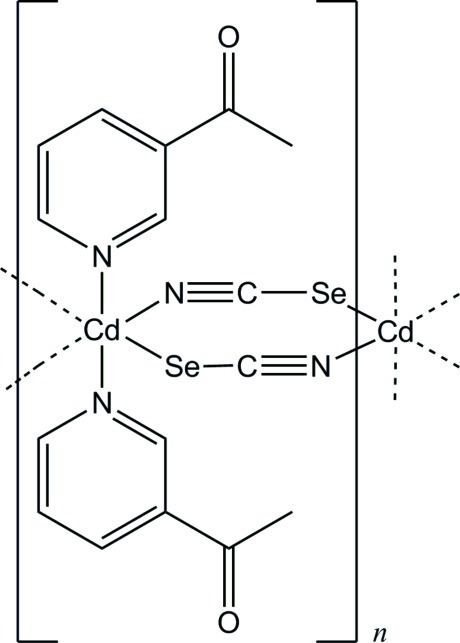


## Experimental

### 

#### Crystal data

[Cd(NCSe)_2_(C_7_H_7_NO)_2_]
*M* *_r_* = 564.63
Monoclinic, 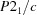

*a* = 5.9447 (3) Å
*b* = 18.7233 (10) Å
*c* = 8.7548 (5) Å
β = 94.020 (4)°
*V* = 972.05 (9) Å^3^
*Z* = 2
Mo *K*α radiation
μ = 4.88 mm^−1^
*T* = 293 K
0.16 × 0.07 × 0.02 mm

#### Data collection

Stoe IPDS-2 diffractometer
Absorption correction: numerical (*X-SHAPE* and *X-RED32*; Stoe & Cie, 2008 ▶) *T*_min_ = 0.667, *T*_max_ = 0.902
17180 measured reflections
2458 independent reflections
2256 reflections with *I* > 2σ(*I*)
*R*_int_ = 0.045

#### Refinement

*R*[*F* ^2^ > 2σ(*F* ^2^)] = 0.031
*wR*(*F* ^2^) = 0.061
*S* = 1.13
2458 reflections
117 parameters
H-atom parameters constrained
Δρ_max_ = 0.47 e Å^−3^
Δρ_min_ = −0.47 e Å^−3^

### 

Data collection: *X-AREA* (Stoe & Cie, 2008 ▶); cell refinement: *X-AREA*; data reduction: *X-AREA*; program(s) used to solve structure: *SHELXS97* (Sheldrick, 2008 ▶); program(s) used to refine structure: *SHELXL97* (Sheldrick, 2008 ▶); molecular graphics: *XP* (Sheldrick, 2008 ▶) and *DIAMOND* (Brandenburg, 2011 ▶); software used to prepare material for publication: *publCIF* (Westrip, 2010) ▶.

## Supplementary Material

Crystal structure: contains datablock(s) I, global. DOI: 10.1107/S1600536812018375/bt5897sup1.cif

Structure factors: contains datablock(s) I. DOI: 10.1107/S1600536812018375/bt5897Isup2.hkl

Additional supplementary materials:  crystallographic information; 3D view; checkCIF report

## Figures and Tables

**Fig. 1.**
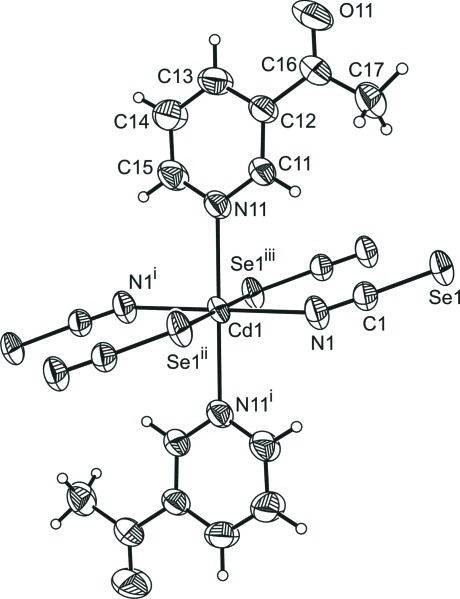
: Crystal structure of the title compound with labelling and displacement ellipsoids drawn at the 50% probability level. Symmetry code: i = -x,-y+1,-z, ii = x-1, y, z, iii = -x+1, -y+1, -z

**Fig. 2.**
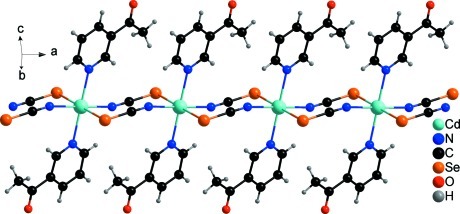
: Crystal structure of the title compound with view of the chains that elongate in the direction of the crystallographic a axis.

## supplementary crystallographic information

### Comment

The title compound was prepared within a project on the synthesis and the magnetic properties of paramagnetic transition metal thio- and selenocyanato coordination polymers in which the metal cations are *µ*-1,3 bridged by the anionic ligands (Näther & Greve, 2003, Boeckmann & Näther, 2010, 2011 and Wöhlert *et al.*, 2011). In this context, also the corresponding compounds based on diamagnetic cadmium are of interest, because they are structural analogs of the paramagnetic compounds. In the course of systematic investigations crystals of the title compound were prepared and characterized by single crystal X-ray diffraction.

In the crystal structure of the title compound, the cadmium(II) cations each are coordinated by two nitrogen atoms of two terminal *N*-bonded 3-acetylpyridine and two nitrogen and two selenium atoms of *µ*-1,3 bridging selenocyanato anions (Fig. 1). The coordination polyhedron of the Cd cations can be described as a slightly distorted octahedra with the Cd cation located on a centre of inversion.

The Cd^2+^ cations are *µ*-1,3 bridged by selenocyanato anions into chains, which elongate in the direction of the crystallographic *a* axis (Fig. 2). The Cd···Cd intrachain distance amounts to 5.9447 (3) Å and the shortest interchain Cd···Cd distance amounts to 8.7548 (5) Å. It must be noted that according to a search in the CCDC database (ConQuest Ver.1.14.2012) (Allen, 2002) coordination compounds based on metal selenocyanates and 3-acetylpyridine are unknown.

### Experimental

Potassium selenocyanate and 3-acetylpyridine were purchased from Alfa Aesar, Cd(NO_3_)_2_.4H_2_O were obtained from Merck. The title compound was prepared by the reaction of 77.1 mg Cd(NO_3_)_2_.4H_2_O (0.25 mmol), 64.8 mg KSeCN (0.45 mmol) and 109 µ*L* 3-acetylpyridine (1.00 mmol) in 1.5 mL H_2_O at RT in a closed 3 ml snap cap vial. After several days colourless needles of the title compound were obtained.

### Refinement

H atoms were positioned with idealized geometry (methyl H atoms allowed to rotate but not to tip) and were refined isotropically with *U*_iso_(H) = 1.2 *U*_eq_(C) for aromatic H atoms (1.5 for methyl H atoms) using a riding model with C—H = 0.93 Å (aromatic) and with C—H = 0.96 Å (methyl).

### Crystal data

**Table:**

[Cd(NCSe)_2_(C_7_H_7_NO)_2_]	*F*(000) = 540
*M**_r_* = 564.63	*D*_x_ = 1.929 Mg m^−^^3^
Monoclinic, *P*2_1_/*c*	Mo *K*α radiation, λ = 0.71073 Å
Hall symbol: -P 2ybc	Cell parameters from 17180 reflections
*a* = 5.9447 (3) Å	θ = 2.2–28.6°
*b* = 18.7233 (10) Å	µ = 4.88 mm^−^^1^
*c* = 8.7548 (5) Å	*T* = 293 K
β = 94.020 (4)°	Needle, colourless
*V* = 972.05 (9) Å^3^	0.16 × 0.07 × 0.02 mm
*Z* = 2	

### Data collection

**Table:**

Stoe IPDS-2 diffractometer	2458 independent reflections
Radiation source: fine-focus sealed tube	2256 reflections with *I* > 2σ(*I*)
Graphite monochromator	*R*_int_ = 0.045
ω scans	θ_max_ = 28.6°, θ_min_ = 2.2°
Absorption correction: numerical (*X-SHAPE* and *X-RED32*; Stoe & Cie, 2008)	*h* = −7→7
*T*_min_ = 0.667, *T*_max_ = 0.902	*k* = −25→25
17180 measured reflections	*l* = −11→11

### Refinement

**Table:**

Refinement on *F*^2^	Secondary atom site location: difference Fourier map
Least-squares matrix: full	Hydrogen site location: inferred from neighbouring sites
*R*[*F*^2^ > 2σ(*F*^2^)] = 0.031	H-atom parameters constrained
*wR*(*F*^2^) = 0.061	*w* = 1/[σ^2^(*F*_o_^2^) + (0.0164*P*)^2^ + 1.1235*P*] where *P* = (*F*_o_^2^ + 2*F*_c_^2^)/3
*S* = 1.13	(Δ/σ)_max_ = 0.001
2458 reflections	Δρ_max_ = 0.47 e Å^−^^3^
117 parameters	Δρ_min_ = −0.47 e Å^−^^3^
0 restraints	Extinction correction: *SHELXL97* (Sheldrick, 2008), Fc^*^=kFc[1+0.001xFc^2^λ^3^/sin(2θ)]^-1/4^
Primary atom site location: structure-invariant direct methods	Extinction coefficient: 0.0074 (5)

### Special details

**Table:**

Geometry. All esds (except the esd in the dihedral angle between two l.s. planes) are estimated using the full covariance matrix. The cell esds are taken into account individually in the estimation of esds in distances, angles and torsion angles; correlations between esds in cell parameters are only used when they are defined by crystal symmetry. An approximate (isotropic) treatment of cell esds is used for estimating esds involving l.s. planes.
Refinement. Refinement of F^2^ against ALL reflections. The weighted R-factor wR and goodness of fit S are based on F^2^, conventional R-factors R are based on F, with F set to zero for negative F^2^. The threshold expression of F^2^ > 2sigma(F^2^) is used only for calculating R-factors(gt) etc. and is not relevant to the choice of reflections for refinement. R-factors based on F^2^ are statistically about twice as large as those based on F, and R- factors based on ALL data will be even larger.

### Fractional atomic coordinates and isotropic or equivalent isotropic displacement parameters (Å^2^)

**Table:**

	*x*	*y*	*z*	*U*_iso_*/*U*_eq_	
Cd1	0.0000	0.5000	0.0000	0.03766 (9)	
N1	0.3333 (4)	0.44388 (15)	0.0928 (3)	0.0478 (6)	
C1	0.5139 (4)	0.43756 (15)	0.1480 (3)	0.0377 (6)	
Se1	0.79466 (5)	0.429324 (18)	0.23721 (4)	0.04545 (11)	
N11	0.0930 (4)	0.60142 (13)	0.1566 (3)	0.0422 (5)	
O11	0.5790 (6)	0.72219 (18)	0.5227 (4)	0.1005 (13)	
C11	0.2729 (5)	0.60423 (17)	0.2585 (3)	0.0430 (6)	
H11	0.3604	0.5633	0.2736	0.052*	
C12	0.3348 (5)	0.66449 (16)	0.3420 (4)	0.0442 (7)	
C13	0.2032 (6)	0.72501 (18)	0.3206 (5)	0.0592 (9)	
H13	0.2402	0.7666	0.3746	0.071*	
C14	0.0168 (7)	0.72283 (19)	0.2183 (5)	0.0675 (11)	
H14	−0.0757	0.7626	0.2034	0.081*	
C15	−0.0302 (6)	0.66083 (17)	0.1387 (4)	0.0533 (8)	
H15	−0.1548	0.6602	0.0683	0.064*	
C16	0.5372 (6)	0.6672 (2)	0.4547 (4)	0.0570 (9)	
C17	0.6815 (6)	0.6032 (2)	0.4794 (4)	0.0632 (10)	
H17A	0.8070	0.6144	0.5503	0.095*	
H17B	0.7360	0.5884	0.3837	0.095*	
H17C	0.5951	0.5653	0.5202	0.095*	

### Atomic displacement parameters (Å^2^)

**Table:**

	*U*^11^	*U*^22^	*U*^33^	*U*^12^	*U*^13^	*U*^23^
Cd1	0.02405 (13)	0.04299 (16)	0.04447 (17)	−0.00160 (10)	−0.00794 (10)	−0.00268 (12)
N1	0.0285 (11)	0.0554 (15)	0.0583 (17)	0.0002 (10)	−0.0059 (11)	0.0068 (12)
C1	0.0305 (13)	0.0398 (14)	0.0425 (15)	0.0013 (10)	0.0002 (11)	0.0028 (11)
Se1	0.02761 (14)	0.0576 (2)	0.04962 (19)	0.00067 (12)	−0.00791 (11)	0.01084 (14)
N11	0.0365 (12)	0.0447 (13)	0.0439 (14)	−0.0023 (10)	−0.0074 (10)	0.0003 (10)
O11	0.088 (2)	0.082 (2)	0.123 (3)	0.0018 (18)	−0.053 (2)	−0.043 (2)
C11	0.0372 (14)	0.0447 (15)	0.0457 (16)	−0.0003 (12)	−0.0075 (12)	−0.0022 (12)
C12	0.0418 (15)	0.0442 (15)	0.0453 (16)	−0.0047 (12)	−0.0064 (12)	−0.0030 (12)
C13	0.067 (2)	0.0412 (17)	0.066 (2)	−0.0009 (15)	−0.0177 (18)	−0.0074 (15)
C14	0.074 (2)	0.0444 (18)	0.079 (3)	0.0125 (17)	−0.029 (2)	−0.0031 (17)
C15	0.0510 (18)	0.0498 (18)	0.056 (2)	−0.0003 (14)	−0.0182 (15)	0.0033 (14)
C16	0.0466 (18)	0.061 (2)	0.062 (2)	−0.0043 (15)	−0.0136 (15)	−0.0132 (16)
C17	0.0481 (19)	0.080 (3)	0.058 (2)	0.0041 (18)	−0.0163 (16)	−0.0063 (19)

### Geometric parameters (Å, º)

**Table:**

Cd1—N1^i^	2.337 (2)	C11—H11	0.9300
Cd1—N1	2.337 (2)	C12—C13	1.382 (5)
Cd1—N11	2.384 (2)	C12—C16	1.502 (4)
Cd1—N11^i^	2.384 (2)	C13—C14	1.376 (5)
Cd1—Se1^ii^	2.8124 (3)	C13—H13	0.9300
Cd1—Se1^iii^	2.8124 (3)	C14—C15	1.373 (5)
N1—C1	1.152 (4)	C14—H14	0.9300
C1—Se1	1.799 (3)	C15—H15	0.9300
Se1—Cd1^iv^	2.8124 (3)	C16—C17	1.481 (5)
N11—C15	1.335 (4)	C17—H17A	0.9600
N11—C11	1.345 (3)	C17—H17B	0.9600
O11—C16	1.206 (4)	C17—H17C	0.9600
C11—C12	1.380 (4)		
			
N1^i^—Cd1—N1	180.00 (12)	C12—C11—H11	118.2
N1^i^—Cd1—N11	89.92 (9)	C11—C12—C13	118.1 (3)
N1—Cd1—N11	90.08 (9)	C11—C12—C16	123.2 (3)
N1^i^—Cd1—N11^i^	90.08 (9)	C13—C12—C16	118.7 (3)
N1—Cd1—N11^i^	89.92 (9)	C14—C13—C12	119.1 (3)
N11—Cd1—N11^i^	180.0	C14—C13—H13	120.5
N1^i^—Cd1—Se1^ii^	86.16 (7)	C12—C13—H13	120.5
N1—Cd1—Se1^ii^	93.84 (7)	C15—C14—C13	118.8 (3)
N11—Cd1—Se1^ii^	87.41 (6)	C15—C14—H14	120.6
N11^i^—Cd1—Se1^ii^	92.59 (6)	C13—C14—H14	120.6
N1^i^—Cd1—Se1^iii^	93.84 (7)	N11—C15—C14	123.7 (3)
N1—Cd1—Se1^iii^	86.16 (7)	N11—C15—H15	118.2
N11—Cd1—Se1^iii^	92.59 (6)	C14—C15—H15	118.2
N11^i^—Cd1—Se1^iii^	87.41 (6)	O11—C16—C17	121.4 (3)
Se1^ii^—Cd1—Se1^iii^	180.0	O11—C16—C12	118.8 (3)
C1—N1—Cd1	159.1 (3)	C17—C16—C12	119.8 (3)
N1—C1—Se1	178.7 (3)	C16—C17—H17A	109.5
C1—Se1—Cd1^iv^	94.39 (9)	C16—C17—H17B	109.5
C15—N11—C11	116.7 (3)	H17A—C17—H17B	109.5
C15—N11—Cd1	119.5 (2)	C16—C17—H17C	109.5
C11—N11—Cd1	123.7 (2)	H17A—C17—H17C	109.5
N11—C11—C12	123.6 (3)	H17B—C17—H17C	109.5
N11—C11—H11	118.2		

Symmetry codes: (i) −*x*, −*y*+1, −*z*; (ii) −*x*+1, −*y*+1, −*z*; (iii) *x*−1, *y*, *z*; (iv) *x*+1, *y*, *z*.
